# Bayesian Analysis Used to Identify Clinical and Laboratory Variables Capable of Predicting Progression to Severe Dengue among Infected Pediatric Patients

**DOI:** 10.3390/children10091508

**Published:** 2023-09-05

**Authors:** Josselin Corzo-Gómez, Susana Guzmán-Aquino, Cruz Vargas-De-León, Mauricio Megchún-Hernández, Alfredo Briones-Aranda

**Affiliations:** 1 Escuela de Ciencias Químicas Sede Ocozocoautla, Universidad Autónoma de Chiapas, Ocozocoautla de Espinosa 29140, Mexico; josselin.corzo@unach.mx; 2 Facultad de Medicina Humana, Universidad Autónoma de Chiapas, Tuxtla Gutiérrez 29050, Mexico; maurimeg@hotmail.com; 3 Escuela Superior de Medicina, Instituto Politécnico Nacional, Ciudad de México 07338, Mexico; lu.aquino128@gmail.com (S.G.-A.); leoncruz82@yahoo.com.mx (C.V.-D.-L.); 4 División de Investigación Hospital Juárez de México, Ciudad de México 07760, Mexico; 5 Hospital de Especialidades Pediátricas, Tuxtla Gutiérrez 29045, Mexico

**Keywords:** naive Bayes classifier, severe dengue, children, data mining, Youden’s J statistic

## Abstract

The current contribution aimed to evaluate the capacity of the naive Bayes classifier to predict the progression of dengue fever to severe infection in children based on a defined set of clinical conditions and laboratory parameters. This case-control study was conducted by reviewing patient files in two public hospitals in an endemic area in Mexico. All 99 qualifying files showed a confirmed diagnosis of dengue. The 32 cases consisted of patients who entered the intensive care unit, while the 67 control patients did not require intensive care. The naive Bayes classifier could identify factors predictive of severe dengue, evidenced by 78% sensitivity, 91% specificity, a positive predictive value of 8.7, a negative predictive value of 0.24, and a global yield of 0.69. The factors that exhibited the greatest predictive capacity in the model were seven clinical conditions (tachycardia, respiratory failure, cold hands and feet, capillary leak leading to the escape of blood plasma, dyspnea, and alterations in consciousness) and three laboratory parameters (hypoalbuminemia, hypoproteinemia, and leukocytosis). Thus, the present model showed a predictive and adaptive capacity in a small pediatric population. It also identified attributes (i.e., hypoalbuminemia and hypoproteinemia) that may strengthen the WHO criteria for predicting progression to severe dengue.

## 1. Introduction

Dengue is transmitted by the bite of an adult female *Aedes aegypti* mosquito infected with one or more of the four dengue virus serotypes [1,2,3,4]. This is the most common arbovirus worldwide, responsible for ~390 million infections and ~96 million symptomatic cases annually [1]. The incidence has shown a tendency to double in the last 30 years and is projected to continue rising in several regions (e.g., Latin America) [2]. The people most affected by dengue are children, adolescents, and young adults [2,3]. It is worrisome that various Latin American countries reported over three million cases of dengue in 2019 [4], with a sharp increase in the number of patients with severe dengue and, consequently, in the dengue-related mortality of children, especially those from five to nine years of age [5,6]. The same trend has occurred concomitantly in the State of Chiapas in southern Mexico [6], although the incidence of severe dengue in the pediatric population in Mexico continues to be below 10% [7].

The acute fever of dengue occurs between two and seven days post-infection. The disease occasionally passes through three phases: febrile, critical, and convalescent [8]. In a few cases, fever leads to extremely grave conditions, especially in the critical phase [9]. The principal causes of death are severe clinical conditions, including shock, severe bleeding, and widespread organ dysfunction [8]. The physiopathological mechanism by which a relatively few individuals infected with dengue progress to a grave illness is multifactorial and poorly understood. It has been proposed that a higher risk of developing severe forms of dengue fever is mainly linked to a combination of viral factors related to the host and the conditions of the endemic area. The latter implies frequent exposure to various serotypes of the disease [10].

A differential diagnosis of dengue is complicated by the fact that the same mosquito vector is responsible for transmitting dengue, Chikungunya, and Zika [11]. Consequently, the proper selection of the profile of clinical variables to be evaluated is critical because it is necessary to evidence factors not only capable of predicting the severity of dengue but also of helping to differentiate this disease from other arboviruses that exist in the pediatric population of an endemic area. The panorama is complicated even further by the influence of comorbidities present in some patients hospitalized with dengue, including cardiovascular, respiratory, and kidney disease [12,13] as well as cirrhosis, immunosuppression, diabetes, and hypertension [14]. These can all favor the severe form of the disease accompanied by the respective complications.

Despite the difficulty involved in opportunely identifying the few cases likely to progress to severe dengue, this task is extremely important because these cases require expert attention in the intensive care unit (ICU). If there is no way to recognize such cases, a large number of patients with dengue are often hospitalized in endemic areas, most only for observation. The result is an excessive burden on the limited resources of the health care system [15,16]. Hence, it is necessary to create a strategy for efficiently classifying dengue patients at the initial stage of the disease into those with and without the probability of progressing to severe dengue. An effective strategy would reduce the patient load on medical services in endemic areas, leading to the provision of better medical attention to the patients most likely to undergo complications as well as the conservation of resources now spent on patients not at risk.

Among the parameters of laboratory tests reported to serve as reliable indicators of the development of severe dengue are a low platelet count [9,15,16], an elevated aspartate aminotransferase (AST) level [17,18], albumin < 35 g/L, and total bilirubin > 17 μmol/L [18]. However, investigation into prognostic indicators is still at an early stage [17,18], and the majority of the studies in the literature lack adequate statistical methodology for determining the sensitivity and specificity of the respective indicators [18,19]. Additionally, some publications have described the limited sensitivity of certain parameters [20].

In addition to establishing a set of parameters that serve as indicators of progression to severe dengue, it is important to develop a novel and flexible tool capable of incorporating such parameters and modeling the effect of covariables in space and time [21]. Bayesian modeling, based on Bayes’ theorem, is a tool that may be instrumental in analyzing small samples of patients to establish prognostic indicators of progression to severe dengue. It can also be utilized to quantify the uncertainty of the resulting estimates [22]. By analyzing the available knowledge of relevant parameters, the Bayes classifier selects a certain set of parameters for a statistical model, which is complemented with observed data. As a result, previous knowledge (expressed as an initial distribution) and observed data are combined in a probability function to make predictions about future events [21].

Research has recently been carried out with Bayesian modeling in other medical areas. This model is reported to have shown the sensitivity of psychological therapy and certain sociodemographic factors as predictors of mental health [23,24]. One study on the Bayesian model explored the capacity of several parameters of laboratory tests (e.g., C-reactive protein, procalcitonin, and fibrinogen) to predict the effect of drug treatments on the recovery of patients with COVID-19 [25]. Furthermore, the model has been employed to examine the distribution of dengue transmission [26].

The aim of the current contribution was to evaluate the capacity of different classifiers to retrospectively predict which infected children (1–14 years of age) would progress to severe dengue. The assessment of pediatric patient files was carried out by machine learning to determine the most suitable model for predicting the development of severe dengue, which was evidenced by the entry of an infected patient into the ICU. Diverse clinical conditions and laboratory parameters were taken into account. The four models tested were the naive Bayes classifier, multilayer perceptron, simple logistic, and LogitBoost. Given that the naive Bayes classifier found factors predictive of severe dengue and showed the best validity, it may represent a reliable tool for identifying those pediatric patients with a greater probability of developing severe dengue and therefore of needing intensive medical care.

## 2. Materials and Methods

The data for this case-control study was gathered by reviewing files of pediatric patients treated from March to December of 2018 in the municipality of Tuxtla Gutiérrez, Chiapas, Mexico. The two public hospitals participating in the study were Dr. Gilberto Gómez Maza Hospital and the Hospital of Pediatric Specialties. The State of Chiapas, in the southernmost part of Mexico, is a dengue endemic region because its tropical climate favors the proliferation of *Aedes aegypti* mosquitoes. A convenience sample was generated for the control and case groups by reviewing 633 files of boys and girls from 1 to 14 years of age. The patients corresponding to the 99 qualifying files all had a diagnosis of dengue confirmed by the RT-PCR (reverse transcriptase-polymerase chain reaction) technique. The case group consisted of patient files evidencing the development of severe dengue, considered when an infected patient required attention in the ICU. The files of the control group indicated the development of the first (feverish) phase of dengue with warning signs of the critical phase but without the need for intensive care. Considering that the incidence of severe dengue is less than 10% in the Mexican pediatric population according to previous reports [7,27], the number of patients in the control group was increased. The purpose was to improve the power of the statistical analysis and reduce the possibility of bias [28].

Dengue was diagnosed with the criteria of the World Health Organization (WHO): confirmation of the infection by the RT-PCR technique, fever > 39 °C at 1–7 days of evolution of the disease, and other signs and symptoms such as headache, retro-orbital pain, myalgia, arthralgia, hemorrhaging, abdominal pain with persistent vomiting, thrombocytopenia (<30.000–100.000/mm^3^), and/or leukopenia (<3000–3500/mm^3^) [8]. The information obtained from the patient files was organized into a database comprised of sociodemographic variables, clinical signs and symptoms of dengue, and the results of laboratory and diagnostic tests (carried out at the time of hospitalization and during the hospital stay).

A descriptive statistical analysis was carried out on IBM Statistics SPSS 21 software by tabulating the frequency of the distinct variables as well as the mean and standard deviation of each one. The variables were scrutinized in relation to a dichotomous classification: whether or not a patient had required attention in the ICU. Significant differences between the two groups were established by examining the mean values of quantitative variables with the Student’s *t*-test and qualitative variables with Fisher’s exact test. The factors potentially predictive of severe dengue were chosen from the attributes available in Weka machine learning software version 3.9.4 (Waikato environment for knowledge analysis, developed by the University of Waikato in New Zealand). Machine learning involves powerful techniques capable of learning from past observations and thus making precise predictions [29]. The selected factors were assessed by classifiers that organized and categorized the data to make predictions about the probability that a given dengue-infected child would or would not require attention in the ICU. The classifiers herein utilized were four algorithms of machine learning: the naive Bayes classifier, multilayer perceptron, simple logistic, and LogitBoost. Once these algorithms were established, ten rounds of cross-validation were performed to provide greater accuracy. Finally, confusion matrices were integrated into each classifier.

The naive Bayes algorithm examines the probability of occurrence of the variable of interest as a function of previous knowledge about associated variables. Since the presence or absence of a certain characteristic is not related to any other characteristic, this technique considers that all the variables contribute independently to the likelihood that the variable of interest will become an existing condition [30].

On the other hand, multilayer perceptron is a type of supervised artificial neuronal network of learning. The pattern of input stimulates the initial layer of neurons of the network to later propagate through the hidden layer or layers and generate the output file. The degree of error found by comparing the output to the expected result leads to an adjustment (reflecting learning) in the weight assigned to the various auto-organized internal connections of neurons capable of recognizing different patterns of data [30]. LogitBoost is an additive logistic regression that applies an impulse (the cost function of logistic regression) in the construction of an additive logit model. This algorithm, classified as weak or basic learning, requires repeated training based on distinct examples to convert a weak prediction into a strong prediction [31]. The fourth algorithm herein evaluated, simple logistic, is designed to predict the value of a result (Y) based on a single entry value (X), generating a model able to predict the probability of success. It creates models of lineal logistic regression [32], which are useful for predicting a dichotomous result such as requiring or not requiring attention in the ICU.

The algorithm “evaluation of complementary subsets” was employed for subset selection by ranking the values of the average merit and average range. The average merit is the mean of the correlation of the likelihood ratio in the ten rounds of cross-validation. The average range refers to the average order of the subsets in each of the ten rounds [33].

Subsequently, the model was examined for sensitivity (the capacity to detect the presence of a condition such as the development of severe dengue), and specificity (the capacity to detect the absence of the same condition). Hence, it was possible to calculate true and false positives as well as true and false negatives, allowing for the determination of the likelihood ratios and the confidence intervals of the measurements of accuracy [34].

The plausible effectiveness of the model in a clinical context was inferred by means of the positive likelihood ratio (LR+) and negative likelihood ratio (LR−). The former is the proportion of true positive patients to false positive patients (sensitivity/1 − specificity), and the latter, the proportion of false negative patients to true negative patients (1 – sensitivity/specificity). For a diagnostic methodology (e.g., the naive Bayes classifier), a higher LR+ and lower LR− indicate greater accuracy [35].

Use was also made of Youden’s J statistic, which summarizes the overall capability of a diagnostic technique to choose the best classifier. This index adds sensitivity and specificity and subtracts 1 (YJ = S + E − 1). The final value (from 0 to 1) represents an inverse relation to the utility of the index.

The current investigation implied a minimal risk for the participants because it is based on information taken from patient clinical files. Nevertheless, the protocol was submitted for approval to the Committee on Ethics and Research of the Secretary of Health of the State of Chiapas (registration # EADIS-16-2020). The data collected were maintained confidential and the anonymity of the patients was guaranteed.

## 3. Results

### 3.1. General Description of the Cases and Controls

Of the 633 patient files reviewed during the period of study, only 99 complied with the inclusion criteria. Among the respective patients, 32 had entered the ICU (19 boys and 13 girls); 67 (46 boys and 21 girls) had not. The participants were organized into age groups: <1, 1–4, 5–9, and 10–14 years old. The children from five to nine years old constituted the group with the highest prevalence for both genders (46.8%) in the case and control groups. The range of ages was similar for both groups.

### 3.2. Comparison between Groups of the Clinical Conditions and Laboratory Parameters

Among the 34 clinical characteristics included, there was a significant difference between the two groups for 19 of them (*p* < 0.05). Three of these were particularly notable in the case group because their significance was much greater than that of the other characteristics (*p* < 0.0001): tachycardia, cold hands and feet, and respiratory failure (Table 1). Of the 14 laboratory parameters examined, only six were significant: the level of albumin, total proteins, leukocytes, neutrophils, direct bilirubin, and total bilirubin (Table 2).

### 3.3. Identification of the Variables Capable of Prognosis of Severe Dengue

**Figure 1:**
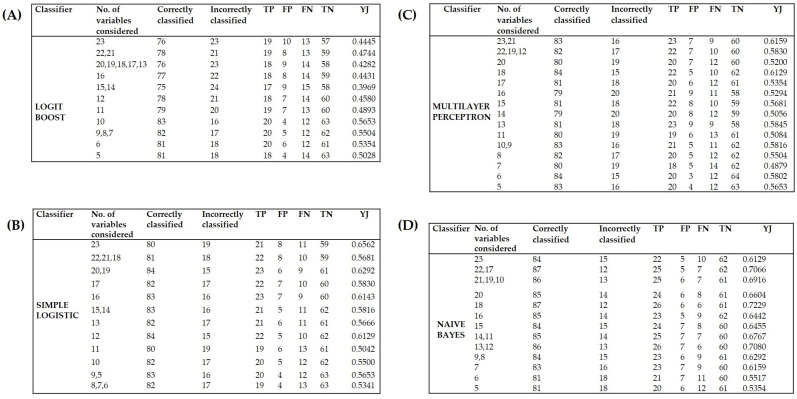
The four algorithms were compared to find the most efficient classifier of cases and controls: (**A**) LogitBoost, (**B**) multilayer perceptron, (**C**) simple logistic, and (**D**) naive Bayes. The best predictive result for each classifier is in bold type. TP, true positive; FP, false positive; FN, false negative; TN, true negative; YJ, Youden’s J statistic.

The classifiers constructed were analyzed with four algorithms: the naive Bayes classifier, multilayer perceptron, simple logistic, and LogitBoost (Figure 1). From 5 to 23 variables were used for each classifier, and the corresponding merit gave greater weight to organ dysfunction, tachycardia, respiratory failure, cold hands and feet, hypoalbuminemia, capillary leak, hypoproteinemia, dyspnea, escape of blood plasma, alterations in consciousness, shock, an increase in the levels of AST/ALT enzymes, disorientation, comorbidities, stupor, hemorrhaging, leukopenia, alterations in the perception of taste, hematemesis, spotted skin, conjunctivitis, pleural effusion, and neutropenia. These variables were eliminated one by one in accordance with their merit (the backward elimination procedure). A record was made of the cases and controls classified correctly and incorrectly, as well as the true positives, false positives, true negatives, false negatives, and overall performance of the models (by means of Youden’s J statistic). When integrating the lowest possible number of attributes [10], the naive Bayes classifier (Figure 1D) proved to be more effective (with YJ = 0.6916) than the other models tested (Figure 1A–C), classifying 86 of 99 cases correctly.

**Figure 2:**
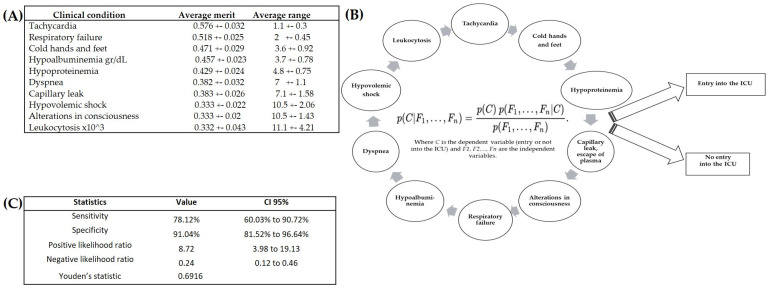
Selection of the best classifier. (**A**) The attributes showing the greatest merit when incorporated into the naive Bayes classifier. (**B**) The model chosen for predicting the entry of patients to the intensive care unit (ICU). (**C**) Evaluation of the model.

The naive Bayes classifier established ten main attributes. The average merit expressed the mean correlation of the likelihood ratio for each one. The average range indicated the mean order in which each attribute was placed in the ten rounds of cross-validation (Figure 2A). The ten best predictive factors of the model consisted of seven clinical conditions (tachycardia, respiratory failure, cold hands and feet, dyspnea, a substantial escape of blood plasma, shock, and alterations in consciousness) and three laboratory parameters (albumin, total protein, and leukocytes) (Figure 2B). The sensitivity of the naive Bayes classifier was 78% and its specificity was 91% (Figure 2C). Of the four methods evaluated, naive Bayes provided the most accurate prognosis, identifying 25 true positive severe dengue cases out of the 32 having required attention in the ICU.

## 4. Discussion

Evidence is herein reported for the first time of the possible usefulness of the naive Bayes classifier for analyzing clinical and laboratory data to find factors that can efficiently predict the progression to severe dengue among pediatric patients infected with the dengue virus. Reliability was achieved through the construction of a Weka software platform (version 3.9.4) of machine learning and the mining of data. Weka is characterized by its analytical computational capability, allowing for the development of an algorithm predictive of probable future cases [36].

Four probabilistic models were presently assessed for their capacity to predict progression to severe dengue based on an analysis of pediatric patient files in two Mexican hospitals in a dengue endemic region. The model that most accurately identified the pediatric patients with dengue that had required attention in the ICU was the naive Bayes classifier. This model produced similar results when fed with different subsets of variables. The best performance was found with a subset of ten predictive factors; seven corresponding to clinical conditions (tachycardia, respiratory failure, cold hands and feet, dyspnea, a substantial escape of blood plasma, shock, and alterations in consciousness) and three to laboratory parameters (hypoalbuminemia, hypoproteinemia, and leukocytosis).

Pone et al. reported lethargy, ascites (abdominal distension), pleural effusion, and hypoalbuminemia as additional markers of the likely progression to severe dengue in hospitalized children [37]. However, the statistical analysis of the latter study was carried out with receiver operating characteristic (ROC) curves, which require a larger quantity of data compared to the analytical method of the current contribution. Furthermore, for each parameter utilized in the ROC curves, the respective reference values must be provided for each age group to allow for a dichotomous classification of the test results [38]. Such reference values may be difficult to obtain in some cases, above all in developing countries with scant research in a given area.

On the other hand, Phakhounthong [39] used the classification and regression tree (CART) analysis to define the principal predictive variables for progression to severe dengue: hypercreatinemia, proteinuria, a decrease in the score on the Glasgow coma scale (indicating alterations in consciousness), and thrombocytopenia. Although the sample (1423 children) was very large, the values of sensitivity and specificity (60.5% and 65%, respectively) were lower than those found presently. Among the many possible reasons for the poor reliability of the model, the likely culprit is the statistical analysis employed to determine the predictive factors, suggesting an opportunity for improvement through the application of Bayesian methodology.

Hypoalbuminemia, herein detected as a predictive factor of severe dengue, is a common denominator described in diverse publications [37,40,41]. It can be associated with capillary leak and the resulting escape of blood plasma, leading to fluid accumulation [39]. Manifestations of fluid accumulation are periorbital puffiness [41], ascites [42], and pleural effusion [37,42], and these conditions have been proposed as predictive factors for severe dengue in children. However, pleural effusion has been shown to be a predictive factor of low specificity (54%) [43]. One study found this factor to be predictive of severe dengue, but the statistical technique (the odds ratio) was weak [42].

In the current contribution, as in previous reports, dyspnea and respiratory failure proved to be two of the main clinical variables capable of predicting progression to severe dengue [44,45]. The identification of these two factors evidences the strength of Bayesian analysis because clinical respiratory indicators could possibly constitute a predictive construct with good validity in the first stages of dengue.

The aforementioned factors exhibit a certain degree of complexity of dengue in a pediatric population, which would likely lead to a lack of specificity for many analytical methods. Thus, a sophisticated tool such as the naive Bayes classifier is required. The most notable difference between frequentist probability and the Bayesian approach is the inclusion of previous knowledge in the latter [46], which, in the present study, allowed for better discrimination between patients who had or had not required attention in the ICU. The naive Bayes classifier was able to identify eight of every ten pediatric patients whose dengue infection required medical attention in the ICU. As can be appreciated, the results emphasize the importance of improving the interpretation of the predictive variables utilized in decision-making, a premise congruent with proposals found in various publications in the literature [23,26,47].

The naïve Bayes classifier established a set of clinical characteristics with the capacity to predict which infected children would progress to severe dengue, without the need for a large sample of hundreds or thousands of people. Hence, Bayesian methodology has the potential for use in similar small pediatric populations in endemic areas. An additional advantage is its adaptability to diverse attributes, including physical, chemical, and biological parameters capable of serving as predictive factors for dengue and other diseases [48,49,50]. For instance, this algorithm is increasingly implemented in the fields of genetics and genomics due to its capacity to handle large sets of data and make predictions about numerous diseases of genetic origin [51]. Moreover, it was adopted by Aswi et al., to examine the relation between the red blood cell count and the length of the hospital stay for dengue patients [26], and those authors found a positive association. Finally, it was utilized to predict the incidence of malaria in African populations by analyzing precipitation, altitude, temperature, the vegetation index, and other factors [52].

The weaknesses of the present study include the limited number of cases and controls and the dispersion of ages of the participants. In part, the small population was a result of the strict implementation of the inclusion criteria. Even though the limited size of the sample did not impede the Bayesian analysis from reaching a good level of reliability, it would be a good idea to apply this predictive model in larger pediatric populations, strictly following sampling rules to better substantiate the current results.

Shock was defined as a predictive factor for severe dengue in the 2009 criteria of the WHO. It is an indicator of a critical loss of plasmatic volume and an increase in the level of leukocytes, especially for patients who were experiencing bleeding. However, the WHO does not consider two factors herein found to be predictive of severe dengue: hypoalbuminemia and hypoproteinemia. Hence, Bayesian analysis could potentially strengthen the WHO criteria by adding important factors for predicting the progression of infected children to severe dengue.

In the southernmost region of Mexico, the area of the present investigation, Chikungunya and Zika are also active viruses. Though they have much lower prevalence rates than dengue, the great similarity of the clinical manifestations produced by all three arboviruses makes an accurate differential diagnosis complex. Thus, it is crucial to follow the recommendations for clinical and laboratory findings provided in the “Guidelines for the Clinical Diagnosis and Treatment of Dengue, Chikungunya, and Zika”, which establish how to handle the three diseases [11]. In the case of dengue, the recommendations can contribute to a decrease in the development of the severe form of the disease.

Future research is needed in which the naive Bayes classifier is applied to diverse scenarios, perhaps including cohort studies, in order to establish a universal algorithm for guiding the clinical management of dengue-infected children living in developing countries. The objective is to classify patients according to their probability of progression to severe dengue, and thus to avoid mortality by focusing hospital resources on the most vulnerable children.

## 5. Conclusions

The naive Bayes classifier identified ten main clinical and laboratory variables that could serve as reliable predictive factors for the progression of infected pediatric patients to severe dengue. These factors coincide with many of those reported previously, thus validating the predictive capacity of the current method of data analysis. The algorithm was adaptable to a small population with complex characteristics, provided good sensitivity and specificity, and even found factors (hypoalbuminemia and hypoproteinemia) not included in the WHO criteria for predicting the progression of infected children to severe dengue. Therefore, with further research to complement and better substantiate the present conclusions, the Bayesian model has the potential to become an effective method for evaluating infected pediatric patients (upon arrival to a hospital in a dengue endemic area) in order to identify those with the probability of progressing to severe dengue.

## Figures and Tables

**Table 1:** Clinical manifestations of patients with severe dengue (the cases) and those in the first (feverish) phase of dengue with warning signs of severe dengue (the controls). Comparison of the clinical variables between cases and controls: *** *p* < 0.0001, based on Fisher’s exact test.

Clinical Variables	Cases (*n* = 32)	Controls (*n* = 67)	Fisher’s Exact Test *p*-Value
Mucosal bleeding	14	13	0.0157
Substantial escape of blood plasma with a weak or undetectable pulse	8	1	0.0004
Tachycardia	15	1	<0.0001 ***
Cold hands and feet	11	1	<0.0001 ***
Respiratory failure	15	2	<0.0001 ***
Hematemesis	8	3	0.0045
Alterations in consciousness	5	0	0.0028
Diaphoresis	5	2	0.0342
Fainting	5	2	0.0342
Conjunctivitis	3	0	0.0316
Cough	7	4	0.0352
Dyspnea	8	1	0.0004
Stupor	4	0	0.0096
Disorientation	4	0	0.0096
Shock	5	0	0.0028
Pleural effusion	7	3	0.0120
Mottled skin	3	0	0.0316
Comorbidities	7	2	0.0047
Hemorrhage	16	13	0.0040

**Table 2:** Laboratory data of patients having severe dengue (the cases) and those manifesting the first (feverish) phase of dengue with warning signs of the severe phase (the controls). The former group consisted of patients that had entered the ICU. A comparison was made between groups of the mean ± standard deviation (x ± SD) of the variables.

Laboratory Variables	Cases (*n* = 32) x (SD)	Controls (*n* = 67) x (SD)	Student’s *t*-Test *p*-Value
Hematocrit	38.58 (8.77)	39.76 (6.07)	0.4377
Hb gr/100 mL	13.34 (3.09)	13.86 (2.10)	0.3277
Platelet × 10^3^	39.63 (58.00)	62.54 (55.92)	0.0626
Albumin gr/Dl	2.95 (0.67)	3.61 (0.50)	<0.0001 ***
Leukocytes × 10^3^	7.77 (4.49)	5.22 (2.70)	0.0007 *
Lymphocytes × 10^3^	1.94 (1.36)	1.72 (1.13)	0.3989
ALT	215.71 (818.36)	53.65 (42.93)	0.1073
AST	757.02 (2841.69)	138.19 (95.34)	0.0765
DB	0.26 (0.40)	0.12 (0.07)	0.0063 **
TB	0.80 (0.53)	0.60 (0.24)	0.0110 *
Alkaline phosphatase u/L	133.73 (62.94)	141.48 (40.86)	0.4636
C-reactive protein mg/Dl	4.67 (7.57)	1.11 (1.53)	0.0003 **
Neutrophils × 10^3^	4.13 (3.26)	2.75 (2.00)	0.0109 *
Total proteins	5.22 (1.05)	6.15 (0.71)	<0.0001 ***

ALT, alanine transaminase; AST, aspartate transaminase; DB, direct bilirubin; TB, total bilirubin; Hb, hemoglobin. * *p* < 0.05, ** *p* < 0.005, *** *p* < 0.0001, based on the Student’s *t*-test.

## Data Availability

The data presented in this study are available on request from the corresponding author. Due to the protection of personal data, the data are not publicly available.

## References

[1] Bhatt S., Gething P.W., Brady O.J., Messina J.P., Farlow A.W., Moyes C.L., Drake J.M., Brownstein J.S., Hoen A.G., Sankoh O. (2013). The global distribution and burden of dengue. Nature.

[2] San Martín J.L., Brathwaite O., Zambrano B., Solórzano J.O., Bouckenooghe A., Dayan G.H., Guzmán M.G. (2010). The Epidemiology of Dengue in the Americas over the Last Three Decades: A Worrisome Reality. Am. J. Trop. Med. Hyg..

[3] Messina J.P., Brady O.J., Golding N., Kraemer M.U.G., Wint G.R.W., Ray S.E., Pigott D.M., Shearer F.M., Johnson K., Earl L. (2019). The current and future global distribution and population at risk of dengue. Nat. Microbiol..

[4] Pan American Health Organization Dengue. https://www.paho.org/data/index.php/en/mnu-topics/indicadoresdengue-en.html.

[5] Dos Santos T.H., Martin J.L.S., Castellanos L.G., Espinal M.A. (2019). Dengue in the Americas: Honduras’ worst outbreak. Lancet.

[6] SINAVE 2018 Panorama Epidemiológico de Dengue 2018. Availaboratory le Online: Panorama Epidemiológico de Dengue 2018—Semana Epidemiológica 52|Secretaría de Salud|Gobierno|gob.mx..

[7] De Antonio R., Amaya-Tapia G., Ibarra-Nieto G., Huerta G., Damaso S. (2021). Incidence of dengue illness in Mexican people aged 6 months to 50 years old: A prospective cohort study conducted in Jalisco. PLoS ONE.

[8] World Health Organization (2009). Dengue: Guidelines for Diagnosis, Treatment, Prevention and Control.

[9] Schaefer T.J., Panda P.K., Wolford R.W. (2023). Dengue Fever. StatPearls.

[10] Rodenhuis-Zybert I.A., Wilschut J., Smit J.M. (2010). Dengue virus life cycle: Viral and host factors modulating infectivity. Cell. Mol. Life Sci. CMLS.

[11] Pan American Health Organization Guidelines for the Clinical Diagnosis and Treatment of Dengue, Chikungunya, and Zika. https://iris.paho.org/handle/10665.2/55867.

[12] Toledo J., George L., Martinez E., Lazaro A., Han W.W., Coelho G.E., Runge Ranzinger S., Horstick O. (2016). Relevance of Non-communicable Comorbidities for the Development of the Severe Forms of Dengue: A Systematic Literature Review. PLoS Neglected Trop. Dis..

[13] Macias A.E., Werneck G.L., Castro R., Mascareñas C., Coudeville L., Morley D., Recamier V., Guergova-Kuras M., Etcheto A., Puentes-Rosas E. (2021). Mortality among Hospitalized Dengue Patients with Comorbidities in Mexico, Brazil, and Colombia. Am. J. Trop. Med. Hyg..

[14] Fonseca-Portilla R., Martínez-Gil M., Morgenstern-Kaplan D. (2021). Risk factors for hospitalization and mortality due to dengue fever in a Mexican population: A retrospective cohort study. Int. J. Infect. Dis..

[15] Lam P.K., Ngoc T.V., Thu Thuy T.T., Hong Van N.T., Nhu Thuy T.T., Hoai Tam D.T., Dung N.M., Tien N.T.H., Kieu N.T.T., Simmons C.P. (2017). The value of daily platelet counts for predicting dengue shock syndrome: Results from a prospective observational study of 2301 Vietnamese children with dengue. PLoS Neglected Trop. Dis..

[16] Dey S.K., Rahman M.M., Howlader A., Siddiqi U.R., Uddin K.M.M., Borhan R., Rahman E.U. (2022). Prediction of dengue incidents using hospitalized patients, metrological and socio-economic data in Bangladesh: A machine learning approach. PLoS ONE.

[17] Nguyen M.T., Ho T.N., Nguyen V.V., Nguyen T.H., Ha M.T., Ta V.T., Nguyen L.D., Phan L., Han K.Q., Duong T.H. (2017). An Evidence-Based Algorithm for Early Prognosis of Severe Dengue in the Outpatient Setting. Clin. Infect. Dis. Off. Publ. Infect. Dis. Soc. Am..

[18] Huy B.V., Toàn N.V. (2022). Prognostic indicators associated with progresses of severe dengue. PLoS ONE.

[19] Thach T.Q., Eisa H.G., Hmeda A.B., Faraj H., Thuan T.M., Abdelrahman M.M., Awadallah M.G., Ha N.X., Noeske M., Abdul Aziz J.M. (2021). Predictive markers for the early prognosis of dengue severity: A systematic review and meta-analysis. PLoS Neglected Trop. Dis..

[20] Djossou F., Vesin G., Elenga N., Demar M., Epelboin L., Walter G., Abboud P., Le-Guen T., Rousset D., Moreau B. (2016). A predictive score for hypotension in patients with confirmed dengue fever in Cayenne Hospital, French Guiana. Trans. R. Soc. Trop. Med. Hyg..

[21] Van de Schoot R., Depaoli S., King R., Kramer B., Märtens K., Tadesse M.G., Vannucci M., Gelman A., Veen D., Willemsen J. (2021). Bayesian statistics and modelling. Nat. Rev. Methods Primers.

[22] Chen D.G., Fraser M.W. (2017). A Bayesian Approach to Sample Size Estimation and the Decision to Continue Program Development in Intervention Research. J. Soc. Soc. Work. Res..

[23] Van Eeden W.A., Luo C., Van Hemert A.M., Carlier I.V.E., Penninx B.W., Wardenaar K.J., Hoos H., Giltay E.J. (2021). Predicting the 9-year course of mood and anxiety disorders with automated machine learning: A comparison between auto-sklearn, naïve Bayes classifier, and traditional logistic regression. Psychiatry Res..

[24] Bone C., Simmonds-Buckley M., Thwaites R., Sandford D., Merzhvynska M., Rubel J., Deisenhofer A.K., Lutz W., Delgadillo J. (2021). Dynamic prediction of psychological treatment outcomes: Development and validation of a prediction model using routinely collected symptom data. Lancet Digit. Health.

[25] Tomasiuk R., Dabrowski J., Smykiewicz J., Wiacek M. (2021). Predictors of COVID-19 Hospital Treatment Outcome. Int. J. Gen. Med..

[26] Aswi A., Cramb S.M., Moraga P., Mengersen K. (2018). Bayesian spatial and spatio-temporal approaches to modelling dengue fever: A systematic review. Epidemiol. Infect..

[27] Gómez-Gómez M., Danglot-Banck C., Huerta Alvarado S.G., García de la Torre G. (2003). El estudio de casos y controles: Su diseño, análisis e interpretación, en investigación clínica. Rev. Mex. Pediatr..

[28] Martínez D., Papuzinski C., Stojanova J., Arancibia M. (2019). General concepts in biostatistics and clinical epidemiology: Observational studies with case-control design. Medwave.

[29] Schapire R.E., Denison D.D., Hansen M.H., Holmes C.C., Mallick B., Yu B. (2003). The Boosting Approach to Machine Learning: An Overview. Nonlinear Estimation and Classification.

[30] Walley W.J., Džeroski S., Denzer R., Schimak G., Russell D. (1996). Biological Monitoring: A Comparison between Bayesian, Neural and Machine Learning Methods of Water Quality Classification. Environmental Software Systems.

[31] Kamarudin M.H., Maple C., Watson T., Safa N.S. (2017). A LogitBoost-Based Algorithm for Detecting Known and Unknown Web Attacks. IEEE Access.

[32] Bandeira A.P. (2015). Aplicação de Rede Neural Artificial para o Reconhecimento do Diabetes Mellitus Gestacional com Marcadores Não-Glicêmicos. Master’s Thesis.

[33] Aler R. Tutorial Weka 3.6.0 Contenidos 2009. https://1library.co/document/y6964ony-tutorial-weka-ricardo-aler-2009.html.

[34] Hernández Rosales D.E. (2017). Modelado de la Capacidad Funcional Articular de la Mano Usando Algoritmos de Inteligencia Artificial en Pacientes con Artritis Reumatoide. Master’s Thesis.

[35] Cifuentes L., Cerda J. (2010). Clinical use of diagnostic tests (Part 2). Clinical application and usefulness of a diagnostic test. Rev. Chil. Infectol..

[36] Witten I.H., Frank E., Hall M.A., Pal C.J. (2011). Data Mining: Practical Machine Learning Tools and Techniques.

[37] Pone S.M., Hökerberg Y.H., de Oliveira R.d.V., Daumas R.P., Pone T.M., Pone M.V., Brasil P. (2016). Clinical and laboratory oratory signs associated to serious dengue disease in hospitalized children. J. Pediatr..

[38] Cerda J., Cifuentes L. (2012). Uso de curvas ROC en investigación clínica: Aspectos teórico-prácticos. Rev. Chil. Infectol..

[39] Phakhounthong K., Chaovalit P., Jittamala P., Blacksell S.D., Carter M.J., Turner P., Chheng K., Sona S., Kumar V., Day N.P.J. (2018). Predicting the severity of dengue fever in children on admission based on clinical features and laboratory oratory indicators: Application of classification tree analysis. BMC Pediatr..

[40] Sangkaew S., Ming D., Boonyasiri A., Honeyford K., Kalayanarooj S., Yacoub S., Dorigatti I., Holmes A. (2021). Risk predictors of progression to severe disease during the febrile phase of dengue: A systematic review and meta-analysis. Lancet Infect. Dis..

[41] Arora S.K., Nandan D., Sharma A., Benerjee P., Singh D.P. (2021). Predictors of severe dengue amongst children as per the revised WHO classification. J. Vector Borne Dis..

[42] Tsheten T., Clements A.C.A., Gray D.J., Adhikary R.K., Furuya-Kanamori L., Wangdi K. (2021). Clinical predictors of severe dengue: A systematic review and meta-analysis. Infect. Dis. Poverty.

[43] Tamibmaniam J., Hussin N., Cheah W.K., Ng K.S., Muninathan P. (2016). Proposal of a Clinical Decision Tree Algorithm Using Factors Associated with Severe Dengue Infection. PLoS ONE.

[44] Wang C.C., Liu S.F., Liao S.C., Lee K., Liu J.W., Lin A.S., Lin M.C. (2007). Acute respiratory failure in adult patients with dengue virus infection. Am. J. Trop. Med. Hyg..

[45] Abhay A.S., Kattoor S., Paul D., Antony T.P. (2020). Clinical course and outcome of dengue fever patients admitted with respiratory manifestations. Pulmon.

[46] Gelman A., Simpson D., Betancourt M. (2017). The prior can often only be understood in the context of the likelihood. Entropy.

[47] Nuzzo R.L. (2017). An Introduction to Bayesian Data Analysis for Correlations. PM R J. Inj. Funct. Rehabil..

[48] Johnson A.E., Pollard T.J., Shen L., Lehman L.W., Feng M., Ghassemi M., Moody B., Szolovits P., Celi L.A., Mark R.G. (2016). MIMIC-III 2016, a freely accessible critical care database. Sci. Data.

[49] Gutierrez G. (2020). Artificial Intelligence in the Intensive Care Unit. Crit. Care.

[50] Greco M., Caruso P.F., Cecconi M. (2021). Artificial Intelligence in the Intensive Care Unit. Semin. Respir. Crit. Care Med..

[51] (2021). Bayesian statistics and modelling. Nat. Rev. Methods Primers.

[52] Sehlabana M.A., Maposa D., Boateng A. (2020). Modelling Malaria Incidence in the Limpopo Province, South Africa: Comparison of Classical and Bayesian Methods of Estimation. Int. J. Environ. Res. Public Health.

